# Trabectedin and Plitidepsin: Drugs from the Sea that Strike the Tumor Microenvironment

**DOI:** 10.3390/md12020719

**Published:** 2014-01-27

**Authors:** Carlos M. Galmarini, Maurizio D’Incalci, Paola Allavena

**Affiliations:** 1 Cell Biology and Pharmacogenomics Department, PharmaMar, Madrid 28770, Spain; E-Mail: cgalmarini@pharmamar.com; 2 Department of Oncology, IRCCS-Istituto di Ricerche Farmacologiche Mario Negri, Milan 20156, Italy; E-Mail: maurizio.dincalci@marionegri.it; 3 Department Immunology and Inflammation, IRCCS Clinical and Research Institute Hu manitas, Rozzano, Milan 20089, Italy

**Keywords:** trabectedin, plitidepsin, tumor-associated macrophages, tumor microenvironment

## Abstract

The prevailing paradigm states that cancer cells acquire multiple genetic mutations in oncogenes or tumor suppressor genes whose respective activation/up-regulation or loss of function serve to impart aberrant properties, such as hyperproliferation or inhibition of cell death. However, a tumor is now considered as an organ-like structure, a complex system composed of multiple cell types (e.g., tumor cells, inflammatory cells, endothelial cells, fibroblasts, *etc.*) all embedded in an inflammatory stroma. All these components influence each other in a complex and dynamic cross-talk, leading to tumor cell survival and progression. As the microenvironment has such a crucial role in tumor pathophysiology, it represents an attractive target for cancer therapy. In this review, we describe the mechanism of action of trabectedin and plitidepsin as an example of how these specific drugs of marine origin elicit their antitumor activity not only by targeting tumor cells but also the tumor microenvironment.

## 1. Introduction

According to the prevailing paradigm of carcinogenesis, normal cells become cancer cells by acquiring multiple genetic mutations in oncogenes or tumor suppressor genes in which respective activation/up-regulation or loss of function served to impart aberrant properties, such as hyperproliferation, blockade of differentiation or inhibition of cell death [1,2,3]. However, a tumor is now considered as an organ-like structure, a complex system composed of multiple cell types (e.g., tumor cells, inflammatory cells, endothelial cells, fibroblasts, *etc.*) all embedded in an inflammatory stroma. All these components influence each other in a complex and dynamic cross-talk, leading to tumor survival and progression [4]. For example, growth factors and cytokines released by non-tumor cells support malignant cell progression and contribute to suppress the immune system inside tumors. Therefore, the microenvironment of a tumor is an integral part of its anatomy and physiology, and functionally, one cannot dissociate this microenvironment from what have traditionally been called “cancer cells” [5,6].

As the microenvironment has such a crucial role in tumor pathophysiology, it represents an attractive target for cancer therapy. There is already a wealth of information about specific cells and molecules in the tumor microenvironment that can be used as targets for cancer therapy at present [7,8]. For this reason, new molecules with antitumor features are looked at with new eyes. Anti-angiogenic therapies, for example, have been shown to improve clinical outcome in patients with different tumor types; similarly, signaling by the extracellular matrix (ECM) and its receptors are also becoming attractive targets of therapy, especially, as it is clear that ECM receptors and growth factor receptors cooperate to maintain tissue specificity. In this review, we describe the mechanism of action of trabectedin and plitidepsin as an example of how drugs of marine origin elicit their antitumor activity by targeting tumor cells but also the tumor microenvironment.

## 2. The Tumor Microenvironment

Organs are composed of parenchymal cells that perform the main organ function and the stroma that gives the supportive framework. In normal tissues, the parenchyma is formed by cells inter-connected by cell-cell junctions that define cell morphology, proliferation, migration, adhesion, differentiation, and cell death [9]. On the other hand, the stroma includes the ECM, fibroblasts, migratory immune cells, and neural elements supported by a vascular network, all within a milieu of cytokines and growth factors. The interstitial matrix is an intricate and highly dynamic network of fibers composed of glycosaminoglycan (GAG)-containing glycoproteins. Different types of fibrous collagen together with fibronectin, hyaluronan, and proteoglycans confer mechanical strength, elasticity and a precise spatial organization to tissues. Organ architecture and function are maintained by dynamic interactions between parenchymal cells and their microenvironment [10]. Both components, cells and stroma, communicate via complex autocrine, juxtacrine, and paracrine mechanisms consisting of soluble molecules, including growth factors, cytokines, hormones, and proteases, insoluble factors, such as ECM components, and direct cell-cell interactions. For example, the ECM contains a wide range of growth factors that are bound in an inactive form to matricellular proteins, but can be rapidly released and activated in case of need, for example during tissue repair.

As any organ, tumors are also composed of cells and stroma. However, both these components are altered. Tumor cells are characterized by the presence of mutations that alter their normal functioning but also their relationship with the surrounding microenvironment. For example, as cancer cells begin to expand, they produce factors that activate myoﬁbroblasts and recruit tumor-associated ﬁbroblasts. These mesenchymal cells, as well as adipocytes, are responsible for many of the tumor-associated changes in the extracellular matrix that promote and foster tumor progression [11]. Normal cells are also active participants that shape the features of tumors. Inﬂammatory cells, including neutrophils, and macrophages, are frequently the ﬁrst immune cells recruited to the tumor, and may be either tumor-promoting or tumor-inhibiting depending on their degree of activation and polarization [5,12]. Another inﬂammatory cell type, the mast cell, is also recruited early and promotes tumor progression by releasing proteases that activate angiogenesis [13]. Myeloid-derived suppressor cells inhibit T-cell activation and promote angiogenesis, cancer cell invasion, and metastasis [14]. Natural killer cells and different types of T cells may have either pro- or antitumor functions, depending on their mode of activation. Immunoglobulins released by B cells promote tumor growth by initiating the inﬂammatory response. Finally, cytokines and chemokines produced by tumor cells, leukocytes and other cell types are key orchestrators of the cancer-related inﬂammation and have long been associated with the recruitment of leukocytes in tumors and therefore with the sustained presence of inﬂammatory cells [15,16,17]. These molecules are involved in important biological processes that govern proliferation and migration of different cells, regulation of innate and adaptive immune responses, and angiogenic and repair programs of the tissues.

On the other hand, the nature of the ECM in the tumor is different from that of the host tissue. The tumor stroma is characterized by a remarkable subversion of the tissue architecture and by a different composition of some ECM components. For instance, whereas the host organ may have been relatively soft and pliable, tumors are often tough and fibrotic. It has recently been shown that these alterations in the mechanical properties of the tissue contribute to the malignant phenotype [18,19,20]. Ultrastructural and immunohistochemical analyses also revealed the up-regulation of several proteins, such as tenascin, decorin, byglican, α-smooth muscle actin, osteopontin, fibulin-1, fibronectin, and the appearance of spliced protein isoforms that are not normally expressed [21,22]. This changed architecture may promote cell invasion by enabling cells to migrate along the collagen ﬁbers or by enhancing integrin signaling [19,23]. Moreover, these matricellular proteins are degraded by specific proteases, such as matrix metalloproteases (MMPs), cathepsins, hyaluronidases, heparanase, elastase, urokinase-type plasminogen activator (uPA), plasmin and others in which activity is generally increased in tumors [24,25]. Finally, tumor blood vessels are also different from those of the normal vasculature as they are irregular and dilated. Furthermore, perivascular cells in tumors are loosely associated with endothelial cells [26,27]. These changes result in abnormal blood ﬂow and leaky blood vessels with extravasation of excess ﬂuid and proteins from the capillaries. The increase in the interstitial fluid pressure generates areas of hypoxia and acidosis inside tumors.

In summary, both normal (fibroblasts, endothelial cells, lymphocytes, macrophages, mast cells, and other cell types) and cancer cells inhabit a complex cellular and matrix ecosystem all of which may interact via juxtacrine and paracrine mechanisms. The constant disruption of homeostasis by proliferating tumor cells produces a chronic inflammatory reaction [28]. However, the classic players in acute inflammation (granulocytes, macrophages, endothelial cells, and fibroblasts) that ordinarily lead to the resolution of a wound through an orderly series of events, instead react paradoxically to the presence of dysfunctional epithelial cells by promoting their survival and replication [28]. This process also includes inflammatory angiogenesis and tissue remodeling. Thus, from this perspective, the microenvironment becomes an integral, essential part of a tumor [29,30].

**Figure 1:**
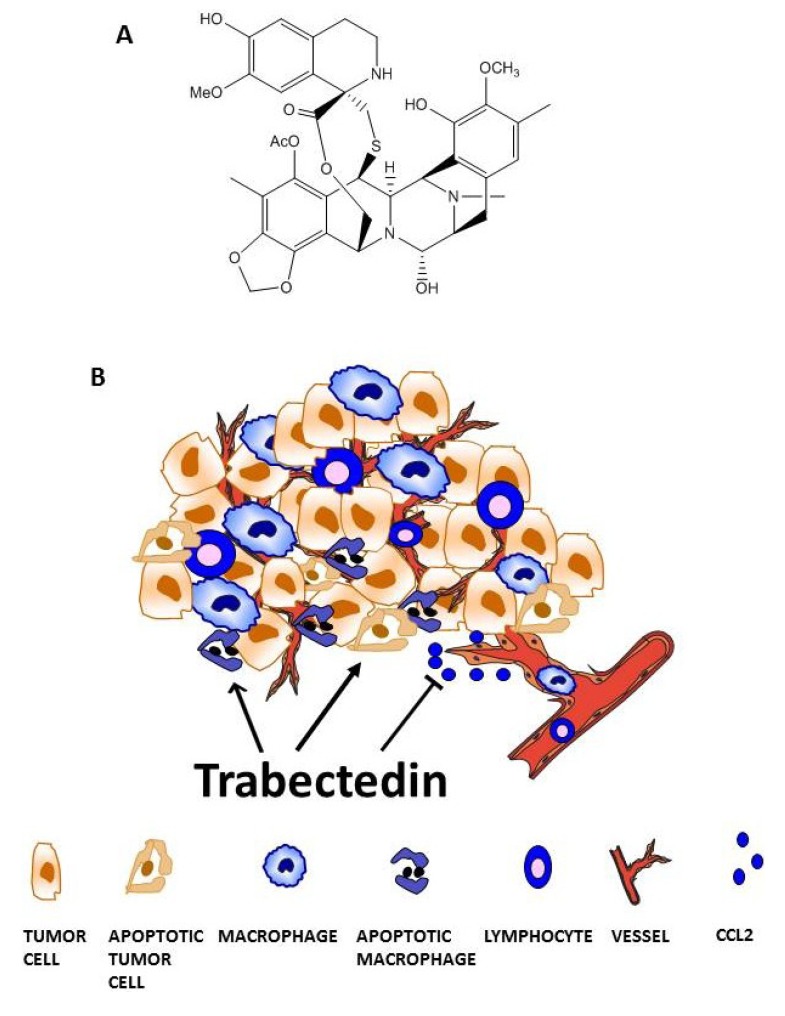
Trabectedin targets tumor-associated macrophages (TAMs). (**A**) Chemical structure. (**B**) Trabectedin acts on the tumor microenvironment by directly affecting monocytes and TAMs or indirectly by inhibiting the secretion of inflammatory mediators involved in different pathophysiological processes, such as inflammatory cell recruitment or tumor angiogenesis.

## 3. Trabectedin Targets Tumor-Associated Macrophages (TAMs)

Trabectedin is an anticancer drug that was originally isolated from the marine Caribbean tunicate *Ecteinascidia turbinata* and now is produced synthetically (Figure 1) [31]. Trabectedin is used for the treatment of soft tissue sarcoma patients and, in combination with pegylated liposomal doxorubicin, for relapsed platinum-sensitive ovarian cancer patients [32,33]. It is a well-tolerated drug; the most common adverse effects observed after administration are reversible neutropenia and reversible AST/ALT elevation. The mechanism of action of trabectedin on tumor cells has been reviewed elsewhere [31]. Of note, the drug shows unique and high-specific inhibition of the transcription process. Transcription inhibition, that affects both the transcribing and the non-transcribing strands, occurs by different mechanisms such as the displacement of specific transcription factors from their promoters, the stabilization of the DNA duplex structure, a direct interaction with RNA Pol II or the induction of the massive degradation of transcribing Pol II [34,35,36,37]. For example, through transcription inhibition, trabectedin modulates the expression of downstream targets, alters tumor biology, and induces the resumption of natural cellular differentiation in sarcomas resulting from the dysregulation of transcription factors, such as EWS-Fli1 fusion protein and FUS-CHOP [31,38,39,40].

In addition to its activity on tumor cells, trabectedin also targets key processes in the biology of tumors, indicating that the drug is more versatile than currently available chemotherapeutic agents. First evidence came from the finding that, among all leukocytes, blood human monocytes and macrophages were hypersensitive to the drug [41]. This finding prompted a series of experiments to understand the exquisite selectivity of trabectedin for mononuclear phagocytes. It was demonstrated that the drug rapidly triggered the activation of caspase 8, downstream of membrane TRAIL receptors (TRAIL-R) [42]. Leukocyte subsets have different sets of TRAIL-R. Monocytes and macrophages mainly express the signaling TRAIL-Rs and are sensitive to trabectedin. In contrast, neutrophils and T lymphocytes preferentially express the non-signaling TRAIL-R (which prevents caspase 8 activation) and are therefore non-susceptible to trabectedin. On the other hand, low, non-cytotoxic concentrations of trabectedin not only inhibit monocyte differentiation into TAMs, but also the production of specific inflammatory mediators, such as CCL2, IL-6, VEGF, and CXC chemokine ligand-8 (CXCL8). This effect was observed particularly in monocytes, TAMs, myxoid liposarcoma cells and ovarian cancer cells [12,31,41]. Other chemokines involved in monocyte recruitment are also transcriptionally affected by trabectedin treatment (e.g., CCL7, CCL3, and CCL14). Importantly, all these effects are not reported for chemotherapeutic agents other than trabectedin (e.g., cisplatin, doxorubicin) [31,41]. Trabectedin also affected the expression of ECM-related genes produced by TAMs and fibroblast, such as fibronectin, osteopontin and matrix-metallo protease-2 (MMP2) or collagen type 1 [43,44]. These results indicate that trabectedin may reduce the high turnover of the tumor stroma.

As described previously, macrophages are a major cellular component of human tumors, where they are commonly termed tumor-associated macrophages (TAMs). These TAMs are derived from monocytes recruited into tumors by chemokines secreted by both malignant and stromal cells [45]. As macrophages, TAMs are versatile cells that are capable of displaying different functional activities. Based on their plasticity, macrophages can be classified in two extreme types: “classical” (or M1) and “alternative” (or M2) [46,47]. After stimulation with interferon gamma (IFN-γ), granulocyte-monocyte-colony stimulating factor (GM-CSF) and tumor necrotic factor-α (TNF-α), M1 macrophages secrete high levels of pro-inflammatory cytokines, such as interleukin-12 (IL-12), interleukin-1 (IL-1), and interleukin-6 (IL-6) and have potent antitumor efficacy [48]. Alternatively, monocytes exposed to interleukin-4 (IL-4) and interleukin-13 (IL-13) become polarized toward the M2-type. This is characterized by higher production of the anti-inﬂammatory cytokine interleukin-10 (IL-10) and low expression of pro-inﬂammatory cytokines and ampliﬁcation of metabolic pathways that can suppress adaptive immune responses [47,49,50]. M2-related activities favor disease progression [51,52,53]. For instance, M2-type TAMs promote tumor angiogenesis by releasing several angiogenic factors, such as vascular endothelial growth factor (VEGF) or platelet-derived growth factor (PDGF) [54]. TAMs also release chemokines (e.g., CCL17, CCL18, CCL22), which increase intratumoral recruitment of lymphoid cells without cytotoxic activity (T-helper 2 lymphocytes; Th2) or with suppressive activity (regulatory T cells; T_reg_) [55]. The impressive array of tumor-promoting functions is consistent with clinical studies showing high macrophage density in many human cancer types to be associated with increased tumor angiogenesis and metastasis, and/or a poor prognosis [56,57,58,59,60,61,62,63,64]. Thus, TAMs are key players of the tumor microenvironment that promote disease progression [12,53,65,66].

The above-mentioned inhibitory activity of trabectedin on TAMs is not only observed *in vitro* but also in various animal tumor models [42,67]. In those models, trabectedin significantly decreased the number of blood monocytes and of tumor-associated macrophages but not of other leukocyte subsets (e.g., neutrophils or lymphocytes) [42]. To prove that the cytotoxic activity on mononuclear phagocytes is an important mechanism of its anti-tumor efficacy, tumor cells resistant to trabectedin *in vitro* were injected into mice [42]. Interestingly, in the *in vivo* setting, trabectedin still showed anti-tumor activity, in spite of the confirmed tumor cell resistance to the drug when exposed *in vitro*. Tumor growth was signiﬁcantly restored after administration of myeloid cells from tumor-bearing untreated mice after each drug treatment cycle. Therefore, macrophage targeting *in vivo* appear to be a key component of the anti-tumor activity of trabectedin. Effects other than macrophage depletion also account for its antitumor efficacy. Using human liposarcoma xenografts grown in nude mice, Trabectedin induces a significant decrease in the expression of relevant tumor mediators such as the chemokines CCL2 and CXCL8, IL-6 or the angiogenic factor VEGF, and an overall effect on the angiogenic network [42,67]. Thus, in addition to direct activity on mononuclear phagocytes, trabectedin reduces the secretion of inflammatory mediators affecting main tumor pathophysiological processes such as the recruitment of circulating monocytes into tumors or angiogenesis [41,42].

The key evidence that trabectedin presents selective activity against monocytes and TAMs finally came from experiments conducted on human samples from patients receiving the drug as part of their therapy. Firstly, a decrease in monocytes was observed within few days after injection of trabectedin in most patients with soft tissue sarcoma [42]. Furthermore, by comparing tumor sections collected before and after neo-adjuvant therapy, a dramatic decrease of macrophage infiltration and vessel network was evidenced, reinforcing the concept that this compound strikes both the neoplastic compartment and the tumor micro-environment. Similarly, monocytes and TAMs from ovarian cancer biopsies were sensitive to trabectedin at low nM concentrations and were much more sensitive than lymphocytes. Moreover, at those concentrations, trabectedin significantly inhibited chemokine (CCL2), inflammatory cytokine (IL-6), and angiogenic factor (e.g., angiopoietin-2, VEGF) production by monocytes, macrophages, and TAMs [31,41].

**Figure 2:**
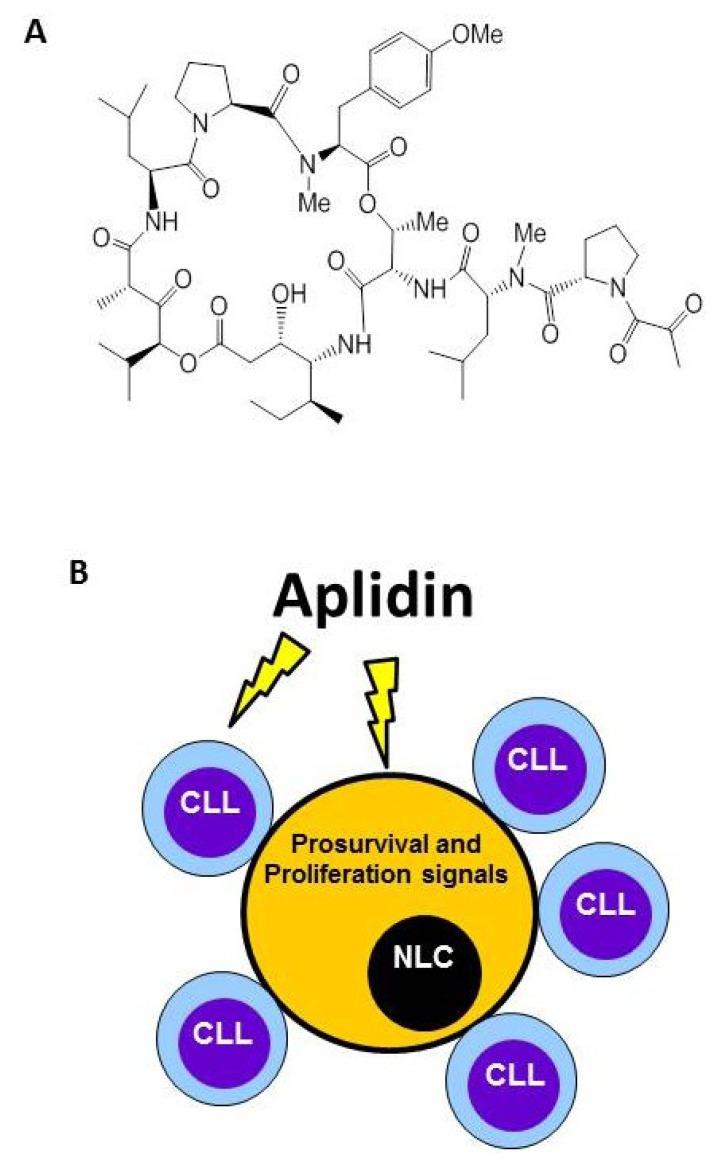
Plitidepsin (Aplidin) targets tumor-associated Nurse-Like cells (NLCs). (**A**) Chemical structure. (**B**) Besides inducing apoptosis of Chronic Lymphocytic Leukemia (CLL) cells, Plitidepsin also acts on monocytes and NLCs. NLCs promote survival of CLL cells by releasing pro-survival factors such as CXCL12.

## 4. Plitidepsin Targets Monocytes and Nurse-Like Cells (NLCs)

Plitidepsin (Aplidin) is a synthetic depsipeptide originally isolated from the tunicate *Aplidium albicans* and now obtained by total synthesis (Figure 2). The compound is currently being evaluated in a Phase III trial for the treatment of refractory/relapsed multiple myeloma patients (clinicaltrial.gov code: NCT01102426). Plitidepsin is a well-tolerated drug. The most common plitidepsin-related adverse events are nausea, fatigue, and myalgia. The mechanism of action of plitidepsin on tumor cells was reviewed elsewhere [68]. Briefly, after interacting with a moderately high-affinity binding site in the cell membrane, plitidepsin led to the rapid activation of Rac1 and the sustained activation of JNK and p38/MAPK that will finally provoke the appearance of a caspase-dependent apoptosis [68,69,70,71]. Results of several studies associated the activation of JNK by plitidepsin with an increased oxidative stress characterized by a rise in the levels of reactive oxygen species (ROS) and a fall in reduced glutathion GSH [71,72]. The oxidative stress induced by Plitidepsin also provokes an endoplasmic reticulum (ER) stress associated to cell death [73]. After cell treatment, plitidepsin triggers the activation of several key molecular components of the classical ER stress-induced unfolded protein response (UPR). These include the proteolytic processing of ATF6 and the alternative splicing of XBP1, as well as the phosphorylation of eIF2α and JNK. Plitidepsin also induces the decrease of CHOP protein levels, due to its rapid degradation by the ubiquitin/proteasome machinery.

In addition to the previously mentioned effects, plitidepsin is also acting on the tumor microenvironment. This is clearly observed on chronic lymphocytic leukemia models (CLL). It was recently published that besides its direct effects on CLL cells, plitidepsin exhibits a potent activity against monocytes and NLCs, a subset of cells derived from peripheral monocytes of CLL patients [74]. The high sensitivity of monocyte and monocytic-derived cells to plitidepsin indirectly affects leukemic cell survival by impairing the delivery of pro-survival signals from these cell populations. As with TAMs in solid tumors, NLCs promote survival of CLL cells and play a crucial role for CLL cell progression. Among other pro-survival factors, NLCs secrete CXCL12, a chemokine that not only induces the migration of CLL cells to lymphoid tissues but also protects them from spontaneous and drug-induced apoptosis in a contact-dependent fashion [75,76]. NLCs can be found in the spleen and secondary lymphoid tissue of patients with CLL [77]. In line with the protective function of NLCs on CLL cells, it was also observed that the previous treatment of isolated NLCs with Plitidepsin impairs the survival of CLL cells. Plitidepsin induced monocyte and NLCs death by triggering apoptosis, as evidenced by early exposure of phosphatidylserine in the outer leaflet of plasma membrane, the activation of caspase-3 and subsequent cleavage of PARP [74]. The production of ROS induced by plitidepsin plays a central role in monocyte death, as drug-induced apoptosis was blocked when preincubating monocytes with ebselen, a compound that increases intracellular GSH levels. It is currently known that ROS and reactive nitrogen species (RNS) regulate the molecular and biochemical pathways responsible for human monocyte survival and that any disbalance in this strict regulation (e.g., induction of oxidative stress), can be detrimental for these cells [78]. In this regard, it was reported that monocytes are more susceptible than lymphocytes to cell death triggered by oxidative stress [79]. These data indicate that, besides its direct antitumor activity on CLL cells, plitidepsin also affects other main drivers of CLL biology, such as monocytic-derived cells.

## 5. Conclusions

In the last decades the concept that the tumor microenvironment is simply a supporting structure for the preservation of tissue architecture has dramatically changed. Indeed, microenvironmental components provide signals affecting cell adhesion, migration, proliferation, differentiation, and death. Monocytic-derived cells are key players of the cancer-related inflammation present at tumor sites. Such a reactive milieu eventually supports tumor cell proliferation and the full-blown development of neo-angiogenesis. There is increasing evidence that successful anti-cancer therapies are not only dependent on the cancer phenotype but also on the normalization of the tumor stroma. In this view, the findings showing that trabectedin and plitidepsin have wider mechanisms of action acting not only on tumor cells but also modifying the whole microenvironment is of great interest and may contribute to the development of new drugs from marine origin as anticancer therapies.

The opportunity to combine direct antiproliferative activity on tumor cells with the capacity to counteract the pro-tumoral properties of the tumor microenvironment is emerging as an especially appealing therapeutic effect of some drugs of marine origin. As reviewed above, the antitumor activity of trabectedin and plitidepsin is not only related to their effects on tumor cells, but also on their ability to affect the tumor microenvironment, in particular monocytic-derived cell (TAMs and NLCs, respectively) and their pro-tumoral functions. Of note, the effects of trabectedin on tumor microenvironment are in line with response patterns evident in several patients, such as tumor shrinkage or delayed response and prolonged stabilization even in the absence of evident tumor shrinkage [80]. Trabectedin can therefore be considered a particularly important antitumor agent with mechanisms of action that make it especially appropriate for targeting key processes in the biology of cancer. Similar considerations can be taken into account for plitidepsin.

To our knowledge, there are no other reports showing this specific activity with other marine-derived drugs. Therefore, whether this is a common feature of marine natural products or a specific mechanism for trabectedin and plitidepsin remains to be elucidated.

## 6. Future Directions

Several questions need to be further addressed concerning the activity of drugs of marine origin on tumor microenvironment. We first need to understand if the activities observed for trabectedin or plitidepsin on the tumor microenvironment can be observed for other marine-derived drugs. A second question is whether these drugs are also affecting other stromal components besides the myeloid-derived compartment. We also need to understand whether the depletion of monocytic-derived cells is similar in all treated patients or if individual variability may result in different therapeutic efficacy. Another important question is whether, by affecting myeloid cells, marine-derived drugs independently influence main pathophysiological pathways of a given tumor. For example, it would be of interest to know if the anti-angiogenic activity of trabectedin is due to a direct effect on the vessel network or is mediated via the reduction of pro-angiogenic cytokines released by TAMs. While it is clear that marine-derived drugs have favorable mechanisms on tumor cells and the tumor microenvironment, additional research into the following actions would be beneficial. All these questions need to be addressed in order to improve the drug discovery and developmental process that would translate into more effective treatments with drugs of marine origin.
